# Contexts That Exclude: Loneliness in North America, Europe, and Africa

**DOI:** 10.1093/geroni/igaa057.2063

**Published:** 2020-12-16

**Authors:** Norah Keating, Vera Roos

**Affiliations:** 1 Swansea University, Summerland, British Columbia, Canada; 2 Optentia Research Focus Area, North-West University, Vanderbijlpark, North-West, South Africa

## Abstract

Loneliness is a key indicator of exclusion from social relations. Its prevalence among older people is of increasing global concern given the powerful impact of loneliness on health and wellbeing. To date, empirical and theoretical advancements in understanding loneliness have been focused on the micro contexts of networks of social relationships surrounding older people. Yet these relationships occur within broader socio-cultural, political and geographical settings that may help us better understand the experiences and manifestations of loneliness. In this presentation we draw on our research on loneliness on three continents. Findings from Canada show how ageing in a foreign land can truncate social connections. Research across Europe reveals differences in values and expectations about social connections. South African findings illustrate the long-term impact of dislocation resulting from political ideologies. We draw together these findings to create an ecological model of contexts that may lead to cumulative disadvantage in social relations.

